# Biomimetic Scaffolds Obtained by Electrospinning of Collagen-Based Materials: Strategies to Hinder the Protein Denaturation

**DOI:** 10.3390/ma14164360

**Published:** 2021-08-04

**Authors:** Giorgia Montalbano, Clarissa Tomasina, Sonia Fiorilli, Sandra Camarero-Espinosa, Chiara Vitale-Brovarone, Lorenzo Moroni

**Affiliations:** 1 Department of Applied Science and Technology, Politecnico di Torino, 10129 Torino, Italy; giorgia.montalbano@polito.it (G.M.); sonia.fiorilli@polito.it (S.F.); 2 Complex Tissue Regeneration Department, MERLN Institute for Technology-Inspired Regenerative Medicine, Maastricht University, Universiteitssingel 40, 6229 ET Maastricht, The Netherlands; c.tomasina@maastrichtuniversity.nl (C.T.); sandra.camarero@polymat.eu (S.C.-E.); l.moroni@maastrichtuniversity.nl (L.M.); 3 POLYMAT, University of the Basque Country UPV/EHU, 20018 San Sebastián, Spain; 4 IKERBASQUE, Basque Foundation for Science, 48009 Bilbao, Spain

**Keywords:** biomimetic scaffolds, electrospinning, type I collagen, hybrid formulations, bone tissue engineering

## Abstract

The use of biomaterials and scaffolds to boost bone regeneration is increasingly gaining interest as a complementary method to the standard surgical and pharmacological treatments in case of severe injuries and pathological conditions. In this frame, the selection of biomaterials and the accurate assessment of the manufacturing procedures are considered key factors in the design of constructs able to resemble the features of the native tissue and effectively induce specific cell responses. Accordingly, composite scaffolds based on type-I-collagen can mimic the composition of bone extracellular matrix (ECM), while electrospinning technologies can be exploited to produce nanofibrous matrices to resemble its architectural organization. However, the combination of collagen and electrospinning reported several complications due to the frequent denaturation of the protein and the variability of results according to collagen origin, concentration, and solvent. In this context, the strategies optimized in this study enabled the preparation of collagen-based electrospun scaffolds characterized by about 100 nm fibers, preserving the physico-chemical properties of the protein thanks to the use of an acetic acid-based solvent. Moreover, nanoparticles of mesoporous bioactive glasses were combined with the optimized collagen formulation, proving the successful design of composite scaffolds resembling the morphological features of bone ECM at the nanoscale.

## 1. Introduction

Biomaterial-based approaches to improve the regeneration of compromised and damaged tissues are increasingly emerging as promising alternatives to overcome the limits presented by the standard surgical and pharmacological treatments [1,2,3]. Especially in the field of bone regeneration applications, the development of scaffolds and biomaterials able to promote and guide the formation of new healthy tissue is considered crucial due to the ever-increasing occurrence of bone loss and damage caused by traumatic or pathological conditions [4,5,6].

In this context, biomimetic and bioactive materials have proved to increase their effectiveness when exploited to design constructs mimicking the structural features of the native extracellular matrix (ECM) [7,8,9]. In the specific case of bone tissue, the natural ECM is a complex hierarchically organized structure mainly consisting of type I collagen fibrils and nanosized hydroxyapatite crystals. Therefore, nano-structured composite scaffolds based on type I collagen can represent biomimetic substrates able to boost the regeneration of bone tissue, reproducing its native chemical and nano-architectural features [9,10].

Besides additive manufacturing technologies, electrospinning represents a promising tool to produce structurally biomimetic scaffolds since it enables the creation of nanofibrous constructs presenting fiber diameters ranging from a few microns to less than 100 nm, thus mimicking the features of the bone ECM at the micro- and nanoscale [11,12]. The overall process consists of the deposition of dry fibers, starting from polymeric solutions that are extruded upon application of an electric field, wherein the deposition and the morphological features of the resulting mats can be partially controlled by the optimization of the process parameters as well as the physico-chemical properties of the material [11,13,14]. Thanks to this manufacturing process, nanostructured scaffolds characterized by a high surface area-to-volume ratio can be realized and possibly exploited as biomimetic matrices as well as efficient delivery platforms upon simple modifications and functionalization of the constructs, as reported in different studies [1,13,15].

In the field of bone tissue engineering, type I collagen, being the main organic component of the bone ECM, represents the most suitable substrate thanks to its well-known high biocompatibility and ability to promote cell adhesion and proliferation [10,16]. The combination of collagen with electrospinning technologies has already proved to be promising in different fields of tissue engineering. In this frame, the study of Rho and coworkers demonstrated that collagen electrospun matrices coated with ECM proteins were very effective as wound-healing accelerators in early-stage wound healing [17]. Moreover, considering the key role of collagen in the composition of native blood vessels, tubular electrospun scaffolds have been explored to actively support vascular regeneration [13].

Despite the chance of creating nanostructured scaffolds reproducing the dimensions of collagen fibrils present in vivo (ranging from 20 to 40 mm), in the use of collagen for electrospinning applications several authors reported critical aspects concerning the potential denaturation of the protein, mainly due to the used organic solvents (e.g., 1,1,3,3,3-hexafluoro-2-propanol (HFP) and 2,2,2-trifluoroethanol (TFE)) and to the process parameters [11,18,19]. On the other hand, the use of non-toxic solvents such as ethanol or PBS solutions often led to the formation of mats with larger and less homogeneous fibers, with high concentrations of deposited salts [20].

Based on these limitations, synthetic polymers are normally preferred thanks to their easier processability, in addition to the enhanced stability and mechanical properties of the resulting scaffolds [15,21]. However, the potential residues of toxic solvents and the synthetic nature of the substrates greatly affect the final biocompatibility and bioactivity of the constructs [15,19].

In the case of naturally derived polymers such as type I collagen, just a few works in the literature documented the use of non-toxic benign solvents, often without reporting evidence on the effective preservation of the physico-chemical properties of the protein [12,18,19]. Moreover, several studies showed morphological features of the scaffolds significantly different on the base of the collagen origin, concentration and solvents used [11,14,18,20].

Despite the combination of type I collagen with other synthetic and natural polymers for electrospinning applications have been already documented, few studies reported the inclusion of inorganic phases for the development of electrospun composite collagen scaffolds [12,13,19,22]. Considering the natural composition of bone, Zhou and coworkers [19] reported the successful electrospinning of collagen and nanohydroxyapatite suspensions, obtaining homogeneous scaffolds characterized by fibers in the range of 600 to 900 nm, with a good dispersion of the inorganic phase even at high concentrations.

Besides hydroxyapatite (HA), the use of mesoporous bioactive glasses (MBGs) for bone tissue engineering has been widely reported thanks to their high bioactive character and their ability to promote the deposition of HA crystals in presence of physiological fluids [23,24,25,26]. Moreover, their high specific surface area can be exploited for the incorporation and/or grafting of a wide range of therapeutic agents and biomolecules, acting as efficient delivery nanocarriers [27,28,29,30]. Despite the combination of collagen and MBGs have been largely explored to produce bone scaffolds with different manufacturing techniques, data regarding the processability of these systems with electrospinning technologies are still lacking [26,31,32,33,34].

In this setting, the present work aimed at exploring the design of biomimetic nanostructured scaffolds based on bovine type I collagen derived from Achilles tendon, further investigating the processability of MBG containing hybrid formulations to obtain composite systems. To this aim, a solution of acetic acid/ethyl acetate/water (AA/EA/H_2_O) and a solution of 40% acetic acid in water (AA) were explored as non-toxic solvents dissolving increasing collagen concentrations and evaluating the influence over the final physico-chemical properties of collagen as well as material processability. The selection of collagen concentrations and solvents were based on previous studies reported in the literature and aimed at preserving the structural properties of collagen, avoiding its denaturation [12,35]. In parallel, the electrospinning conditions have been optimized exploiting a plate collector, to obtain homogeneous and defect-less scaffolds, morphologically assessed by means of scanning electron microscopy (SEM). Upon optimization of the collagen solution, MBG particles were incorporated to confirm the processability of hybrid formulations with electrospinning technologies in order to obtain more biomimetic composite scaffolds.

## 2. Materials and Methods

### 2.1. Preparation of Collagen-Based Materials for Electrospinning

To develop and optimize collagen-based formulations suitable for electrospinning applications, bovine type I collagen powders derived from Achilles tendon (BOV-COL) (Kensey Nash Corporation, Greater Philadelphia Area, Great Lakes, Northeastern US) were dissolved at different concentrations ranging between 12 and 25 wt% in two different solvents: a solution of acetic acid/ethyl acetate/water (AA/EA/H_2_O) with a ratio of 40:30:30 and a solution of 40% acetic acid in water (AA). To promote the proper dissolution of collagen powders considering the high concentrations, the solutions were stirred at 4 °C overnight and used immediately after preparation.

After the optimization of collagen concentration and solvent, nanoparticles of mesoporous bioactive glasses (MBG_SG), prepared following a sol-gel route previously optimized by the authors (Supplementary Material) [36,37], were used to obtain bioactive hybrid formulations. After complete dissolution of collagen powders in 40% AA at 4 °C, MBG_SG nanoparticles were sonicated in the same solvent using a horn sonicator (Sonoplus Ultraschall-Homogenisation HD 2200, 20 kHz ± 500 Hz frequency, 50% amplitude) for 6 min and subsequently incorporated into the collagen solution to obtain final concentrations of 5 wt%. The resulting suspension was stirred for 1 h at room temperature to obtain a homogeneous system and immediately used for electrospinning tests.

### 2.2. Circular Dichroism (CD)

CD analyses were performed with a JASCO J-815 circular dichroism spectropolarimeter, (Jasco, Mary’s Court, Easton, MD, USA) equipped with a Xe arc lamp, to record data in the far-UV spectral range. CD spectra resulted from the average of 3 scans recorded for each sample at 50 nm/sec scanning rate. The measurements were carried out using a quartz circular cuvette with a path length of 0.1 mm in the 180–260 nm wavelength range. Collagen samples recovered after dissolution in the different solvents (AA/EA/H_2_O, 40% AA) and after electrospinning were prepared using a concentration of 0.1 mg/mL in distilled water. All spectra were corrected after acquisition using the correspondent solvent medium as baseline (distilled water) and analyzed at room temperature (20 °C).

### 2.3. Rheology

All the rheological tests were performed using a DHR-2 controlled stress rotational Rheometer (TA Instruments, Waters, New Castle, IN, USA) equipped with a parallel plate geometry with a diameter of 20 mm and a Peltier plate system to constantly control the system temperature. Flow ramp tests at 23 °C were conducted to investigate the viscosity of the different collagen solutions and the final suspension over a wide range of shear rates (0.01–1000 s^−1^).

### 2.4. Electrospinning Tests

The electrospinning tests were performed using a custom-made electrospinning setup equipped with a temperature and humidity control system. A plate collector and a spinneret tip of 21 G (0.8 mm internal diameter) were used for all the tests in order to obtain randomly oriented fibrous mats.

After preparation, the different collagen-based formulations were left 30 min at room temperature before loading into 5 mL syringes.

Electrospinning tests were conducted at a distance between spinneret tip and collector of 10–15 cm (working distance) with voltage and flow rate ranging from 18.5 to 20 kV and 0.05 to 0.15 mL/h, respectively, and keeping a constant temperature of 23 °C at 40% humidity. The resulting electrospun matrices were removed from the collector and left to dry overnight at room temperature before analysis.

### 2.5. Scaffold Characterisation

#### 2.5.1. Scanning Electron Microscopy (SEM) and Dispersive X-ray Analysis (EDX)

For investigation of fiber diameter, mesh morphology and particle distribution, samples of electrospun scaffolds were punched into 12 mm disks and gold coated using a Cressington Sputter Coated 180 auto and then imaged using a Jeol JSM-IT200 InTouchScope scanning electron microscope (JEOL, Peabody, MA, USA) at V = 10 keV.

For the analysis of scaffold composition, scaffolds were studied under the energy dispersive X-ray analyzer (EDX) coupled to the Jeol JSM-IT200 microscope.

#### 2.5.2. Fourier Transform Infrared Spectroscopy (FTIR)

FTIR spectroscopy (Perkin Elmer Spectrum 1000) (JEOL, Peabody, MA, USA) was performed in the 4000–500 cm^−1^ range at a spectral resolution of 0.09 cm^−1^ and accumulation of 32 scans on the optimized electrospun collagen scaffolds with and without MBG_SG particles, compared with non-treated bovine collagen powders.

For the tests, the electrospun scaffolds were dried overnight, peeled from the aluminum foil, and used for measurement.

## 3. Results and Discussion

### 3.1. Assessment and Electrospinning of Different Collagen Formulations

The choice of solvent and polymer concentration plays a critical role in the scaffold fabrication alongside electrospinning parameters such as flow rate, voltage, and collector distance. Solvents normally used for electrospinning applications have been found to significantly influence the native physico-chemical properties of collagen, while the process conditions mainly affect the final scaffold morphology and characteristics [13].

The electrospinning of natural polymers has reported different complications due to their high viscosity and low solubility, often requiring the addition of synthetic polymers or the use of organic toxic reagents, responsible for the evident denaturation of the protein native structure [11,12].

Based on these considerations, more environmentally benign and less harsh solvents were explored to obtain nanostructured collagen electrospun scaffolds. Accordingly, acidic solvent mixtures were evaluated to allow collagen solubilization at high concentrations as well as the native structure preservation.

As the starting point and based on previous studies in the literature [12], a solution of acetic acid (AA), ethyl acetate (EA) and distilled water (dH_2_O) with a ratio of 40:30:30 (AA/EA/dH_2_O) was prepared for collagen solubilization. The addition of ethyl acetate was tested to potentially increase the conductivity of the solution while supporting the formation of a stable electrostatic jet. In parallel, limiting the water component may be beneficial to improve the spinnability and avoid high surface tension [12,13,18].

Collagen powders were dissolved at 120 mg/mL (12%BOV-COL in AA/EA/dH_2_O) and 200 mg/mL (20%BOV-COL in AA/EA/dH_2_O) in the previously prepared solvent (AA/EA/dH_2_O) kept at 4 °C, to evaluate the influence of increasing concentrations over the final material spinnability.

For the electrospinning tests, the jet stabilization was obtained, for each formulation, varying the applied voltage and the material flow between 18.5–20.0 kV and 0.05 to 0.15 mL/h, respectively, using a plate collector and keeping constant conditions of temperature and humidity.

**Figure 1:**
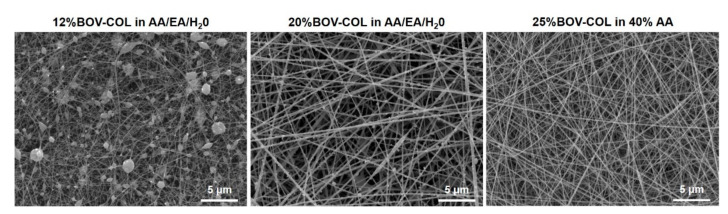
SEM images of scaffolds obtained from the electrospinning of 12%BOV-COL in AA/EA/dH_2_O, 20%BOV-COL in AA/EA/dH_2_O and 25%BOV-COL in 40% AA.

The resulting scaffolds were then collected and analyzed by means of scanning electron microscopy (SEM) in order to define their morphological features. (Figure 1).

The morphological assessment of scaffolds obtained by processing collagen solutions in AA/EA/dH_2_O, revealed that the use of a lower concentration of collagen (12 wt%) resulted in meshes presenting a large number of beads of variable size embedded into the fibers scattered throughout the scaffold structure.

When the collagen concentration was increased up to 20 wt%, the number of defects and beads decreased and the resulting scaffolds showed fibers from 100 to 400 nm (Figure S2B), characterized by a randomly oriented matrix.

As reported in the literature [38,39], the formation of beaded fibers during electrospinning of polymers is considered a quite common phenomenon, mainly due to the instability of the polymeric jet. Specifically, higher values of viscosity and net charge density of the jet as well as reduced surface tension have been observed to decrease the formation of beads, as confirmed by our results. Therefore, not only the concentration and physico-chemical properties of the polymer but also the selected solvents play a key role in the final morphology of the electrospun fibers.

Besides the large presence of defects in the final matrix, the use of AA/EA/dH_2_O as collagen solvent led to a poor stability of the system, where a phase separation was observed after 1 h of material processing (Figure S1B). This effect was tentatively ascribed to the low solubility of bovine collagen powders in the prepared solvent mixture. Therefore, based on the potential negative effects over scaffold fabrication in the long term, an alternative solvent consisting in 40% acetic acid in water was assessed for further investigation.

Considering the high solubility of collagen in acetic acid and the demonstrated beneficial effects of increased collagen concentrations over the final fibrous structure [11,14,15,35], a 25 wt% collagen concentration was chosen and dissolved in 40% acetic acid (40% AA). Upon complete dissolution, the 25%BOV-COL in 40% AA formulation was loaded in the syringe (Figure S1C) and electrospun at 20 kV with a flow rate of 0.05 mL/h and 15 cm of collector distance to obtain a stable material jet.

As reported in Figure 1, the resulting scaffolds presented homogeneous fibers characterized by a diameter of about 135.9 ± 20.7 nm (Figure S2C), comparable to the morphological features observed when processing the 20%BOV-COL in AA/EA/dH_2_O formulation. Moreover, the collagen system proved to be stable during the process and no beads were observed in the resulting structure.

**Figure 2:**
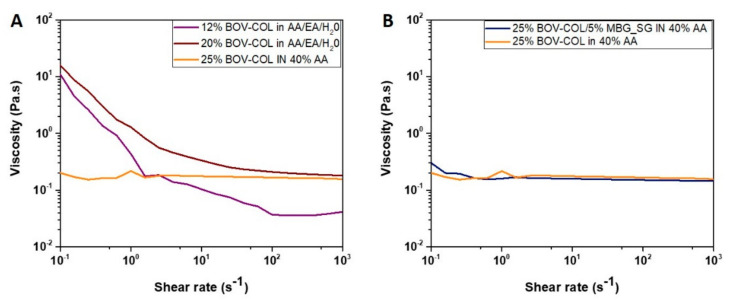
Comparison between flow ramps obtained from 12%BOV-COL in AA/EA/dH_2_O, 20%BOV-COL in AA/EA/dH_2_O and 25%BOV-COL in 40% AA (**A**), and flow ramps of 25%BOV-COL in 40% AA and 25%BOV-COL/5%MBG_SG in 40% AA (**B**).

To further investigate the physico-chemical properties of the developed formulations and the potential influence of the two solvent systems, the variation of the viscosity values was registered by means of rheological analyses, as shown in Figure 2.

As shown in the graph presented in Figure 2A, 12 and 20% BOV-COL in AA/EA/H_2_O systems showed a shear thinning behavior and higher values of viscosity at almost zero-shear conditions. On the contrary, a higher concentration of collagen (25 wt%) dissolved in 40% AA showed a Newtonian behavior, characterized by constant and lower values of viscosity in the wide range of applied shear rates.

These results confirmed the key role of acetic acid in promoting the dissolution of collagen molecules, associated with lower viscosity values, as previously reported [5]. Accordingly, the higher values of viscosity registered for collagen-dissolved AA/EA/H_2_O suggested a lower solubilization of the protein.

### 3.2. Assessment of the Preservation of the Supramolecular Structure of Collagen

The high solubility of collagen molecules in acid solutions and organic solvents is due to the absence of covalent bonds between triple helices of collagen after extraction from native tissues [14]. Higher solubility of collagen is normally preferred to produce more stable and homogeneous solutions, thus allowing the electrospinning and the achievement of homogeneous sub-micrometric fibers, also supported by high polymeric concentrations.

However, as previously reported, the use of organic solvents and strong acids has been suggested to hinder the natural folding of collagen into triple helix, almost absent in the resulting electrospun scaffolds [11,12,13,14].

In this frame, the influence of AA/EA/H_2_0 and 40% AA solvents over the final supramolecular structure of collagen was investigated by means of circular dichroism spectroscopy, observing the potential decrease in the triple helical fraction.

**Figure 3:**
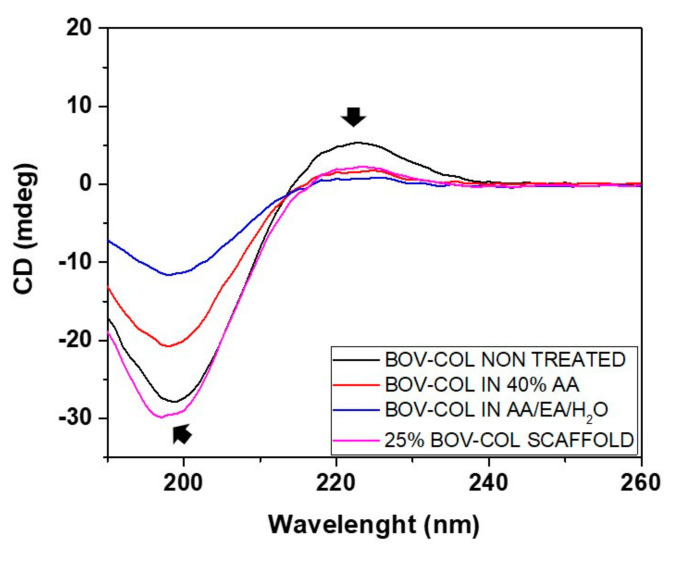
Circular dichroism analysis performed on native collagen (BOV-COL NON-TREATED), collagen reconstituted after dissolution in AA/EA/H_2_O (BOV-COL in AA/EA/H_2_O), collagen reconstituted after dissolution in 40% AA (BOV-COL in 40%AA) and collagen scaffolds obtained by processing 25% BOV-COL in 40% AA (25% BOV-COL SCAFFOLD).

The influence of the solvents alongside the electrospinning process was defined comparing native bovine collagen powders with collagen reconstituted after dissolution in both AA/EA/H_2_O and 40% AA, and electrospun scaffolds obtained by the processing of 25% COL in 40% AA, as shown in Figure 3.

The spectrum derived from original collagen powders (black curve) displayed a sinusoidal pattern with a deep negative peak at about 198 nm, a cross-over around 215 nm and an evident positive peak at 220 nm, indicating the presence of a triple helical configuration [20]. In detail, the negative peak is characteristic of randomly arranged α-chains while the positive one corresponds to the triple helix structure, whose intensity decreases upon denaturation [11,14].

In parallel, collagen reconstituted after dissolution in 40% AA (BOV-COL in 40%AA) and electrospun scaffolds obtained by processing 25% BOV-COL in 40% AA (25% BOV-COL SCAFFOLD) exhibited similar trends, comparable with that reported by native collagen, showing an evident negative peak at about 198 nm and a slightly weakened positive band at 222 nm. Moreover, left shifts of the curves, normally related to a non-triple-helical conformation, were not detected, further confirming the previous comments.

On the contrary, collagen reconstituted after dissolution in AA/EA/H_2_O solvent (BOV-COL in AA/EA/H_2_O) lacked the positive peak, as normally occurs for gelatin and denatured collagen, due to the disruption of the triple helix conformation and the progressively dissociation into the randomly coiled α-chains [11].

These results evidenced that the use of 40% AA and the further processing with electrospinning led to a greater preservation of the ultramolecular structure of collagen compared to the systems obtained from AA/EA/H_2_O and other solvents, in accordance with previous studies reported in the literature [11,14].

Based on the reported results and the demonstrated enhanced processability of the formulation, 25% BOV-COL in 40% AA was selected for further investigation regarding the combination with inorganic phases, in order to obtain biomimetic composite scaffolds for bone tissue engineering.

### 3.3. Electrospinning of Composite Scaffolds

The introduction of inorganic phases in the collagenous system is crucial to obtain biomimetic structures, mimicking the composite nature of bone tissue while reproducing its architecture at the micro- and nanoscale, exploiting electrospinning techniques. Accordingly, the use of MBGs in the form of spheroidal nanoparticles was explored, with the aim to enhance the overall bioactivity of the final constructs.

According to the literature, mesoporous bioactive glasses have largely proved their ability to stimulate bone regeneration, wherein the high surface area and regular nano-porosities also make these materials suitable as efficient delivery platforms for a variety of active molecules and therapeutic agents [28,36,40].

Following the optimized sol-gel route reported by the authors in a previous work (Supplementary Material) [36], the resulting MBG_SG particles presented a spherical morphology, size ranging between 100 and 500 nm, specific surface area between 400 and 500 m^2^/g, and mesopores of 2–4 nm. After preparation, MBG_SG particles were combined with the previously optimized collagen formulation; to this aim, the most straightforward strategy is to disperse them into the pre-formed collagen solution.

As preliminary assessment and based on previous studies reporting the processing of polymeric composite systems [13,15,19], MBG_SG were mixed with collagen to reach a final concentration of 5 wt%, obtaining a homogeneous 25% BOV-COL/MBG_SG in 40% AA hybrid formulation.

According to the rheological analysis reported in Figure 2B, the collagen-based suspension showed a Newtonian behavior, registering low and constant values of viscosity, as observed for the 25% BOV-COL in 40% AA solution. Therefore, the introduction of MBG_SG did not alter the rheological properties of the system, suggesting a good dispersion of the nanoparticles as well as weak interactions between the inorganic and the organic phases [19].

**Figure 4:**
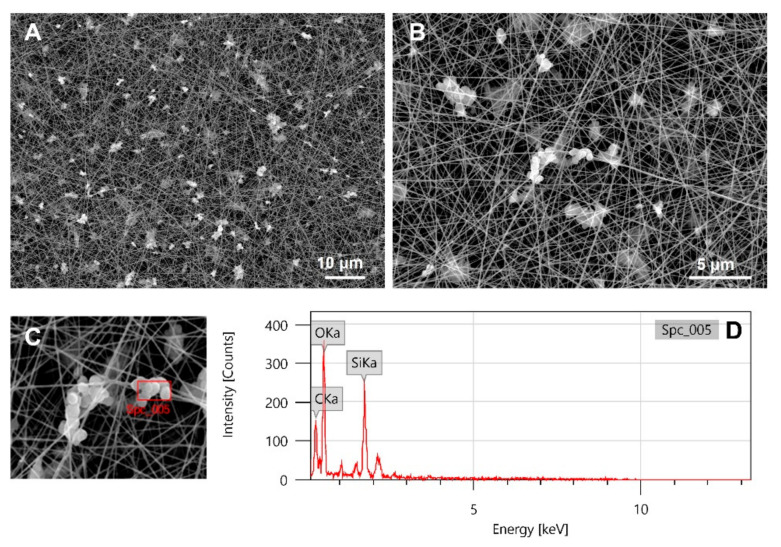
SEM images (**A**–**C**) and EDX analysis (**D**) of 25% BOV-COL/MBG_SG scaffolds.

Despite the similar rheological properties, the electrospinning of the 25% BOV-COL/MBG_SG in 40% AA system showed better performances at different process parameters compared to the other collagen solutions. Specifically, the scaffolds were fabricated with 20 kV voltage and flow rate of 0.1 mL/h, at 12 cm working distance. The resulting scaffolds were then collected and morphologically analyzed by means of SEM, as presented in Figure 4.

As visible from the SEM images, the resulting scaffolds were characterized by randomly oriented homogeneous fibers of about 120 ± 10 nm (Figure S2D), which was comparable to the fibers formed during the electrospinning of collagen alone. The particles looked overall uniformly distributed and small aggregates of about 3 μm were present throughout the scaffold, confirming that the dispersion process successfully limited their aggregation.

The collected scaffolds were further analyzed by means of Energy-dispersive X-ray spectroscopy (EDX) to confirm the composition and the distribution of nanoparticles throughout the matrix, discriminating from inorganic particles and potential collagen beads, as shown in Figure 4D.

At variance with the spectrum of 25% BOV-COL scaffolds reported in Figure S3, EDX spectrum of the 25% BOV-COL/MBG_SG scaffold revealed the presence of MBG_SG particles, evidencing the contribution of silica by Si and O peaks, whereas the peak of C clearly arose from the collagenous matrix.

**Figure 5:**
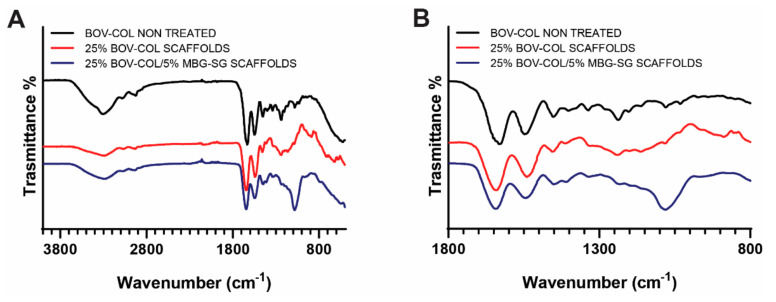
ATR-FTIR of 25% BOV-COL and 25% BOV-COL/MBG_SG scaffolds compared to native collagen (BOV-COL NON-TREATED): whole spectrum (**A**) and focus between 1800 and 800 cm^−1^ (**B**).

The reported results were supported and confirmed by FTIR analysis, where the preservation of the collagen physico-chemical features was also investigated by comparing the spectrum of original collagen powders with the curves obtained from 25% BOV-COL and 25% BOV-COL/MBG_SG scaffolds, as represented in Figure 5.

The spectrum of non-treated bovine collagen powders displayed the characteristic peaks at 3305 and 2922 cm^−1^ related to amide A and B bands, respectively, while peaks at 1628, 1548 and 1237 cm^−1^ were attributed to the amide I, amide II and amide II absorptions [18,19].

As summarized in Table 1, the spectra of 25% BOV-COL and 25% BOV-COL/MBG_SG scaffolds depicted similar patterns when compared with original collagen, without significant alterations in peak intensity of shifts. The slight shift of amide I in 25% BOV-COL and 25% BOV-COL/MBG_SG scaffolds was ascribed to potential residuals of acetic acid, normally detected at about 1700 cm^−1^. However, the absence of an evident peak at 1700 cm^−1^ suggested an almost total removal of the solvent during the electrospinning process [12].

According to previous studies [11,12,14], significant shifts of amide I and II towards lower wavelength as well as the loss of amide III peak at about 1240 cm^−1^ indicate the presence of unfolded structures related to the partial or total denaturation of the protein. As reported by previous studies [41], the amide I band resonates at different wavenumbers depending on the secondary structure of the protein. Specifically, β-turns resonate from 1660 to 1700 cm^−1^, α-helices from 1645 to 1659 cm^−1^, irregular structures from 1640 to 1644 cm^−1^ and β-sheets from 1620 to 1640 cm^−1^, where shifts to lower frequencies are indicative of higher hydrogen bonding potential. Therefore, the detection of amide III peak in both 25% BOV-COL and 25% BOV-COL/MBG_SG spectra, as well as the absence of significant shifts for amide I and II, suggested the good preservation of the integrity of collagen triple helices structures, confirming the results obtained by circular dichroism analysis.

To further confirm this result, the ratio between peaks at 1240 cm^−1^ (amide III) and 1450 cm^−1^ was calculated to be about 0.9 for both native and electrospun collagen, wherein values around 1 are indicative of a triple helical structure and values around 0.5 suggests the protein denaturation [41]. As additional observation, 25% BOV-COL/MBG_SG presented a less evident band for amide III at 1231 cm^−1^ due to the contribution of Si-O-Si band centered at about 1082 cm^−1^ of MBG_SG nanoparticles. The similar values registered for the different spectra also indicated that the addition of MBG_SG and the applied conditions did not destabilize the secondary structure of collagen.

The characterization performed on the developed electrospun composite scaffolds evidenced the successful achievement of homogeneous matrices reproducing the architectural feature of the native extracellular matrix of bone at the nanoscale. Moreover, the developed strategies proved to have less impact on the physico-chemical properties of type I collagen, preserving the supramolecular structure, in contrast with other studies reported in the literature [11,14].

Based on these results and the well-known high bioactive character of constructs consisting of type I collagen and MBGs [24], future studies will be focused on the optimization of the composite scaffolds in terms of mechanical and biological features, with the aim to boost their potential in bone tissue regeneration.

## 4. Conclusions

The outcomes achieved in this study confirmed the processability of collagen-based formulations for the design of biomimetic nanostructures scaffolds by means of electrospinning technologies and highlight that a strict control of polymeric concentration, solvents and process conditions is essential to obtain homogeneous systems and stable material jets.

The proper selection of solvents and electrospinning conditions proved to highly affect the preservation of the physico-chemical properties of collagen as well as the final morphological features of the scaffolds. In this frame, 40% acetic acid solution was used to dissolve high concentrations of bovine collagen powders (25 wt%) to obtain a final system able to be processed by means of electrospinning and forming homogeneous fibrous scaffolds with fibers measuring about 100 nm in diameter. On the contrary, the use of acetic acid (AA), ethyl acetate (EA), and distilled water (dH_2_O) with a ratio of 40:30:30 and lower concentrations of collagen (12 and 20 wt%) led to the formation of less homogeneous fibrous scaffolds presenting a great number of defects.

The electrospinning of collagen thus evidenced the promising advantage of replicating the nano-architecture of the bone tissue, characterized by collagen fibrils ranging between tens of nanometers up to few microns. Alongside the morphological aspects, the introduction of bioactive reinforcing inorganic phases was exploited to boost the potential regenerative effect of the final scaffolds. Based on the well-known bioactive character of mesoporous bioactive glasses and the limited data in the literature presenting their combination with collagen for electrospinning applications, nano-sized mesoporous bioactive glasses (5 wt%) were successfully incorporated into the collagen system previously optimized and composed of 25 wt% collagen dissolved in 40% acetic acid.

The resulting scaffolds presented a uniform nano-fibrous pattern and the homogeneous distribution of inorganic particles throughout the collagen matrix, while the further characterization of the constructs confirmed the preservation of the physico-chemical properties of collagen.

The promising results presented in this study thus pave the way for the design of new composite bioactive scaffolds based on type I collagen and MBGs, able to mimic the features of the native bone ECM and without altering the physico-chemical properties of collagen, crucial to obtain an effective biological response. Accordingly, based on the well-known biocompatibility and bioactivity of both collagen and bioactive glass particles, future studies will be focused on the investigation of scaffold–cell interactions, with the aim to better understand the potential influence of the developed systems over the bone tissue regeneration process.

## Figures and Tables

**Table 1:** FTIR characteristic peaks of collagen, 25% BOV-COL and 25% BOV-COL/MBG_SG scaffolds.

Material	Amide A (cm^−1^)	Amide B (cm^−1^)	Amide I (cm^−1^)	Amide II (cm^−1^)	Amide III (cm^−1^)	Si-O-Si (cm^−1^)
BOV-COL (non-treated)	3305	2922	1628	1548	1237	-
25%BOV-COL SCAFFOLDS	3288	2930	1640	1540	1239	-
25%BOV-COL/5%MBG_SG SCAFFOLDS	3288	2932	1643	1544	1231	1082

## Data Availability

The data presented in this study are openly available in ZENODO at https://doi.org/10.5281/zenodo.5156181 (accessed on 4 August 2021).

## Supplementary Materials

The following are available online at https://www.mdpi.com/article/10.3390/ma14164360/s1, Figure S1: Image showing the 20%BOV-COL in AA/EA/H2O solution immediately after preparation (A) and the phase separation after 1 hour of processing (B), compared to 25% BOV-COL in 40% AA solution immediately after preparation (C) and after 1 hour of processing (D)., Figure S2: Fiber diameter distribution of 12%BOV-COL in AA/EA/H2O (A), 20%BOV-COL in AA/EA/H2O (B), 25%BOV-COL in 40% AA (C) and 25% BOV-COL/MBG_SG (D). Histograms have been obtained by the analysis of SEM images, on 100 fibers for each material. Figure S3: EDS analysis performed on 25% BOV-COL scaffolds.
