# Supplementary material for: Biomimetic Scaffolds Obtained by Electrospinning of Collagen-Based Materials: Strategies to Hinder the Protein Denaturation
Source: Materials (Basel). 2021 Aug 4;14(16):4360. doi: 10.3390/ma14164360 (PMC8400144; doi:10.3390/ma14164360)
Supplement: Supplementary file 1 [file materials-14-04360-s001.zip › materials-1304815 - supplementary.pdf]

materials

MDPI

Supplementary Materials

Biomimetic Scaffolds Obtained by Electrospinning of Collagen-Based Materials: Strategies to Hinder the Protein Denaturation

Giorgia Montalbano [1,†], Clarissa Tomasina [2,†], Sonia Fiorilli [1], Sandra Camarero-Espinosa [2,3,4], Chiara Vitale-Brovarone [1,*] and Lorenzo Moroni [2]

1. Department of Applied Science and Technology, Politecnico di Torino, 10129 Torino, Italy; giorgia.montalbano@polito.it (G.M.); sonia.fiorilli@polito.it (S.F.)
2. Complex Tissue Regeneration Department, MERLN Institute for Technology-Inspired Regenerative Medicine, Maastricht University, Universiteitssingel 40, 6229 ET Maastricht, The Netherlands; c.tomasina@maastrichtuniversity.nl (C.T.); sandra.camarero@polymat.eu (S.C.-E.); l.moroni@maastrichtuniversity.nl (L.M.)
3. POLYMAT, University of the Basque Country UPV/EHU, 20018 San Sebastián, Spain
4. IKERBASQUE, Basque Foundation for Science, 48009 Bilbao, Spain

* Correspondence: chiara.vitale@polito.it

† Equal contribution.

Synthesis of nano-sized mesoporous bioactive glasses (MBG_SG)

Mesoporous bioactive glasses containing (Ca/Si = 15/85) were synthetized exploiting a base-catalyzed sol–gel method following the protocol previously reported by the authors [34]. In details, the calculated amount of cetyltrimethylammonium bromide (CTAB 98%, Sigma Aldrich, Italy) were dissolved in 140 mL of ethanol, 50 mL of double distilled water (ddH_2O) and 25 mL of NH_4OH (Ammonium hydroxide solution, Sigma Aldrich, Italy) for 30 min under stirring. A second solution containing tetraethyl orthosilicate (TEOS, Tetraethyl orthosili-cate, reagent grade 98%, Sigma Aldrich, Italy) and 50 mL of ethanol was prepared and stirred for 30 min. Then, the TEOS solution was added dropwise into CTAB solution and the resulting solution was stirred for 20 min. Calcium nitrate tetrahydrate ($Ca(NO_3)_2 \cdot 4H_2O$, 99%, Sigma Aldrich, Italy) was subsequently added and stirred for 10 min. A further amount of TEOS was then added drop by drop to the solution and stirred for 2 h. The powder was collected by centrifugation (Hermle Labortechnik Z326) at 10,000 rpm for 3 minutes and washed three times with ddH_2O, once with 50% ethanol and once with absolute ethanol. The final precipitate was dried at 70 °C overnight and calcined at 600 °C for 5 hours with a heating rate of 1 °C min-1 in a Carbolite 1300 CWF 15/5, in order to remove CTAB. All the reagents were purchased from Sigma Aldrich (Italy) and used as received.

Phase separation in 20%BOV-COL in AA/EA/H_2O solution

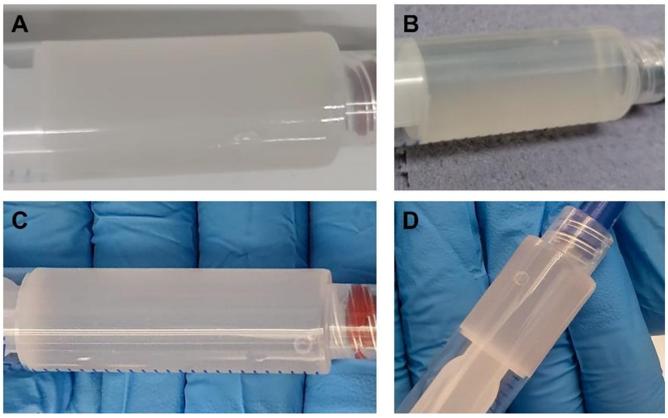

Figure S1. Image showing the 20%BOV-COL in AA/EA/H_2O solution immediately after preparation (**A**) and the phase separation after 1 hour of processing (**B**), compared to 25% BOV-COL in 40% AA solution immediately after preparation (**C**) and after 1 hour of processing (**D**).

Histograms of fiber diameter distribution.

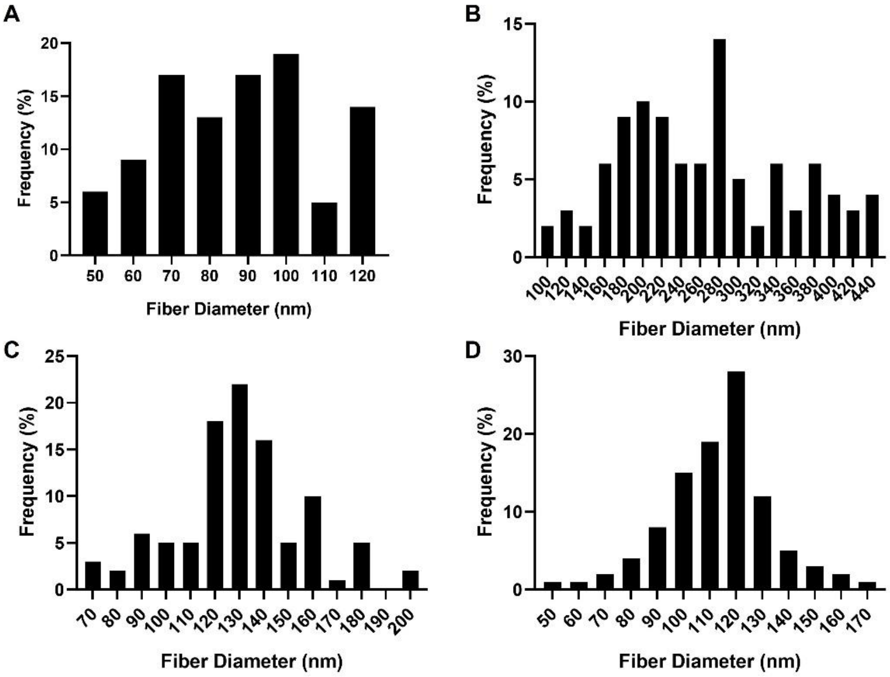

Figure S2. Fiber diameter distribution of 12%BOV-COL in AA/EA/H_2O (**A**), 20%BOV-COL in AA/EA/H_2O (**B**), 25%BOV-COL in 40% AA (**C**) and 25% BOV-COL/MBG_SG (**D**). Histograms have been obtained by the analysis of SEM images, on 100 fibers for each material.

EDS analysis on 25% BOV-COL scaffolds.

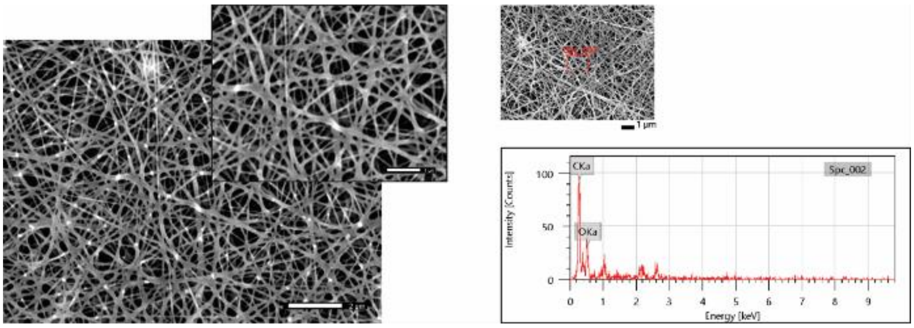

Figure S3. EDS analysis performed on 25% BOV-COL scaffolds.
